# Clinical efficacy and safety of Ganshuang granules as an adjuvant treatment for chronic hepatitis B liver fibrosis: A protocol for systematic review and meta analysis

**DOI:** 10.1097/MD.0000000000022692

**Published:** 2020-10-09

**Authors:** Shaoqian Zeng, Yefang Liu, Cen Jiang, Baixue Li, Li Wen, Quansheng Feng

**Affiliations:** a Chengdu University of Traditional Chinese Medicine; b No. 3 Affiliated Hospital of Chengdu University of TCM (West District), Chengdu Pidu District Hospital of TCM, Chengdu, Sichuan, China.

**Keywords:** adjuvant treatment, chronic hepatitis B liver fibrosis, Ganshuang granules, protocol

## Abstract

**Background:**

Chronic hepatitis B liver fibrosis is significant public concern. Ganshuang granules (GSG) are used to treat liver fibrosis for a long time. The aim of this study is to synthesize related data to explore efficacy and safety of GSG as an adjuvant treatment for chronic hepatitis B liver fibrosis.

**Methods:**

Electronic database were used to identify related studies. We chose PubMed, China Knowledge Network Infrastructure, China Biomedical Database, Wan Fang Data, VIP Database, EMBASE, and Cochrane Library as retrieval tool. Two independent individuals conducted the publication selection, data extraction, data assessment. Any problems between 2 researchers will be resolved by a third reviewer through negotiation. RevMan 5.3 (The Cochrane Collaboration, Copenhagen, Denmark) software will be used for data analysis.

**Results:**

This study will systematically detect the efficacy and safety of GSG for treating chronic hepatitis B liver fibrosis.

**Conclusion:**

This study will provide scientific evidence to explorer whether GSG are efficacy and safety in treating chronic hepatitis B liver fibrosis.

**PROSPERO registration number:**

INPLASY202090027

## 1 Introduction

Chronic hepatitis B virus infection, a global distribution disease, is severe issue due to the adverse sequelae-related morbidity and mortality.^[1–2]^ People often acquire this infection via birth or person-to-person transmission. The estimated global prevalence of chronic HBV infection was 3% to 5% in 2016, with 257 million people chronically infected.^[3]^ Vaccination shows effective in prevention of infection and virus carriage.^[4]^ With the declining prevalence of infection globally, this virus remain a serious threat in many countries and regions.^[5]^ Liver fibrosis is a main adverse sequelae of chronic hepatitis B hepatitis, which some patients leads to cirrhosis, even hepatocellular carcinoma. Liver fibrosis is considered to be reversible.^[6]^ For chronic hepatitis B liver fibrosis, the most effective treatment is antiviral therapy. Antiviral therapy can decrease the risk for developing cirrhosis and progression to cancer, even can reverse liver fibrosis.^[7]^ Due to the low cure rates, life-long antiviral therapy is significant for most patients. However, anti-fibrogenic agents including resveratrol, vitamin E, silymarin still lack sufficient evidence. To date, there are no biochemical drugs for liver fibrosis approved by the FDA.

Traditional Chinese medicine (TCM) is used for treating chronic hepatitis B liver fibrosis for decades.^[8]^ Because of syndrome differentiation, treatment, and multi-target pharmacological effect, TCM demonstrates an unique advantage in treating chronic hepatitis B liver fibrosis.^[9]^ As a complementary and alternative medicine, TCM provide a new methods to anti- chronic hepatitis B liver fibrosis. Ganshuang granules (GSG) is a prepared prescription drug to treat liver fibrosis. This drug is composed of Dangshen (*Codonopsis pilosula*), Chaihu (*Nasturtium officinale*), Baishao (*Paeonia lactiflora*), Danggui (*Angelica sinensis*), Fuling (*Wolfiporia extensa*), Baizhu (*Atractylodes macrocephala*), Zhike (Hedgehog), Pugongyin (Dandelion), Huzhang (Giant knotweed), Xiakucao (Selfheal), Danshen (*Salvia miltiorrhiza*), Taoren (Peach kernel), Biejia (Turtle shell). In China, GSG combined with antiviral therapy is indicated for chronic hepatitis B liver fibrosis. Recent study shows that GSG inhibits stimulation of liver stellate cells through by inhibiting mammalian target of rapamycin.^[10]^ Another experiment suggests that the mechanism of anti-liver fibrosis of GSG is suppressing regulatory T cells.^[11]^

GSG in combination with antiviral treatment is widely used for treating chronic hepatitis B liver fibrosis in China. However, the efficacy and safety of GSG are still unclear. Herein, we performed this research to evaluate effective and safety of GSG. We hope this work will contribute to the clinical application of GSG as adjuvant treatment for chronic hepatitis B liver fibrosis.

## 2 Methods

### 2.1 Inclusion criteria

#### 2.1.1 Types of study

Any randomized controlled trials involving efficacy and safety of GSG for treating chronic hepatitis B liver fibrosis will be included.

#### 2.1.2 Participants

In this study, any subject with chronic hepatitis B liver fibrosis will be included, regardless of age and gender.

#### 2.1.3 Interventions and comparison

The treatment group are treated with GSG combined with entecavir. The control group are oral administration of entecavir in any dosage form (tablets, dispersions, capsules).

### 2.2 Outcomes

The primary outcomes of this study are hyaluronic acid, type IV collagen, laminin, and pre-type-iii collagen, which are the 4 indicators of liver fibrosis. The additional outcomes of this study include ALT, AST, and HBV-DNA negative conversion rates.

### 2.3 Search strategy

This research is conducted in accordance with the Preferred Reporting Items for Systematic Reviews and Meta-analyses Statement guidelines.^[12]^ We conduct this study through by searching Web of Science, PubMed, China Knowledge Network Infrastructure, China Biomedical Database, Wan Fang Data, VIP Database, EMBASE, and Cochrane Library. The above database will be retrieved from its establishment to July 2020. The terms or key words will be searched singly or in varying combinations, as shown in Table 1.

**Table 1:**
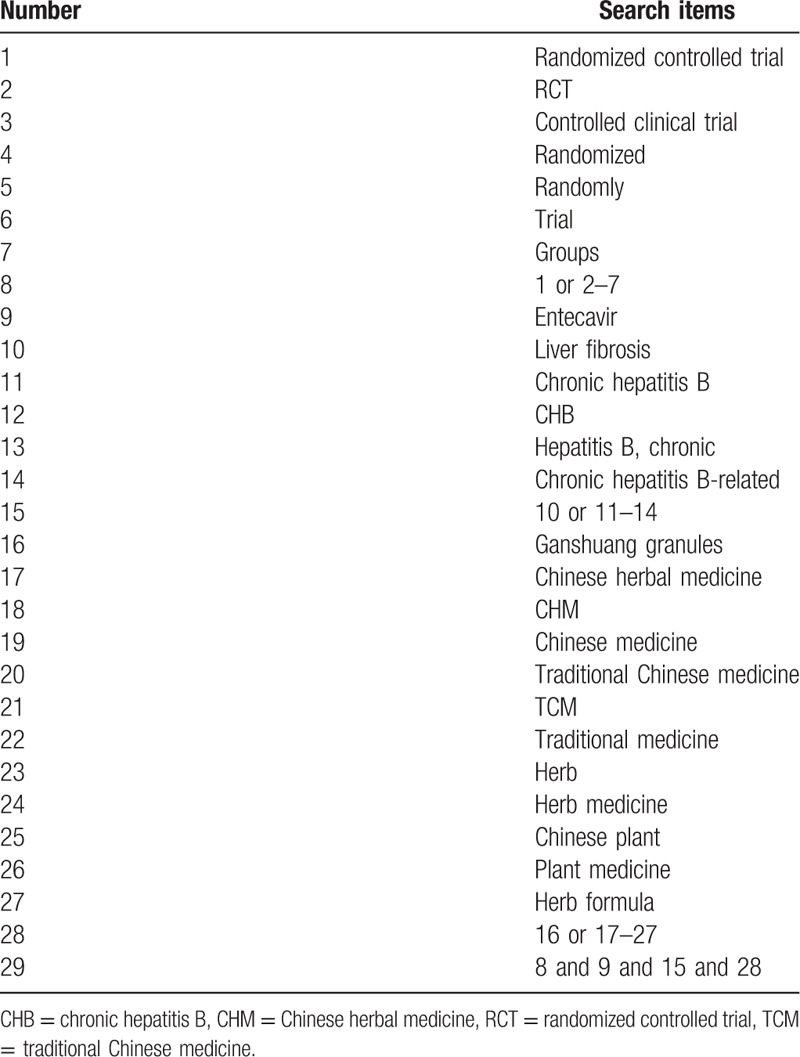
Search strategy used in PubMed database.

### 2.4 Studies selection and data extraction

**Figure 1:**
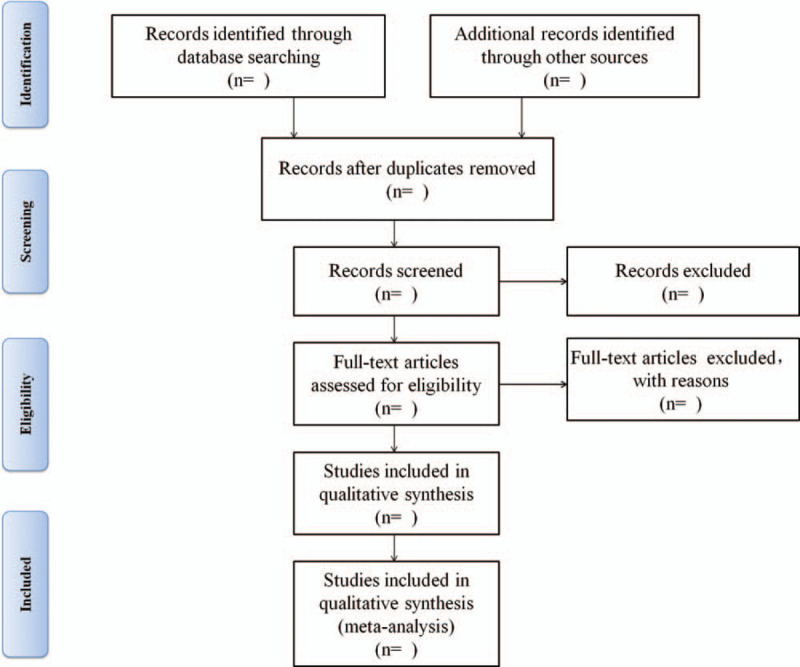
Flow chart of the study selection process.

Two authors (SQ Zeng and YF Liu) independently screen the titles and abstracts to select potentially relevant manuscripts to meet the inclusion criteria. Duplicate and irrelevant articles will be removed. Any divergence will be settled by consultations. Selected papers will be reviewed by third reviewer. Two authors (SQ Zeng and YF Liu) independently check the titles and abstracts of papers to evaluate whether the papers meet the eligible criteria. All papers included will be investigated as full text. If a given paper is in doubt, the final decision will be made by a third reviewer. The content extracted from publications are author, journal, publication year, study setting; participant demographics and baseline characteristics (Fig. 1).

### 2.5 Risk of bias assessment

Methodological quality of included trials will be assessed by Cochrane risk of bias tool.^[13]^ Every study will be assessed the risk of bias. We will divide each study into 3 grades: low risk, high risk and unclear risk. An information sheet will list the results of quality evaluation.

### 2.6 Data synthesis

Data analysis will be conducted by RevMan 5.3 software (The Cochrane Collaboration, Copenhagen, Denmark). Weighted mean difference (WMD) will be used for continuous outcomes. Risk ratio will be adopted for dichotomous results. Heterogeneity across studies will be checked by using I^2^ test. I^2^ < 50% or *P* > .10 suggests heterogeneity across studies, and a fixed-effects model will be conducted for pool analysis. If I^2^ > 50% or *P* < .10, the pooled effect sizes will be calculated by a random-effects mode. The confidence interval will be set at 95%. The publication bias will be visually assessed by funnel plots.

### 2.7 Analysis of subgroup

If required, a subgroup analysis will be performed based on the different study characteristics, study quality, and outcome measurements.

### 2.8 Sensitivity analysis

Stability of merger results will be checked by sensitivity analysis. If any low-quality trials exist, we will remove it.

### 2.9 Ethics and dissemination

This study involves publications only and will not collect any information from patients. Thus, the ethical approval is not required.

This study will be submitted on a peer-reviewed journal.

## 3 Discussion

Chronic hepatitis B virus infection remains an urgent medical challenge worldwide. With active viral replication, this chronic infection may continue to progress, and liver fibrosis, cirrhosis, hepatocellular carcinoma may occur. Chronic hepatitis B liver fibrosis is prevalent in Asia-Pacific countries and regions.^[5]^ Antiviral therapy is fundamental to clinical practice. However, potent anti-fibro genic agents are still lacking.^[14]^ Therefore, clinicians intend to utilized other methods to address this issue when implementing antiviral therapy. In China, TCM has been treating illnesses for a long time. In TCM theory, liver fibrosis mainly caused by blood stasis in the liver. It is believed that GSG can promotes blood circulation in the liver to slow down liver fibrosis progression. Consequently, GSG combined with antiviral therapy is often used in clinical practice for treating chronic hepatitis B liver fibrosis. This protocol is drafted to investigate efficacy and safety of GSG, which is an adjunctive drug in this illness. We expect this study can provide clinical evidence to guide the GSG usage.

## Author contributions

**Conceptualization:** Cen Jiang, Baixue Li.

**Data curation:** Shaoqian Zeng, Yefang Liu.

**Formal analysis:** Shaoqian Zeng, Yefang Liu.

**Funding acquisition:** Quansheng Feng, Cen Jiang, Li Wen.

**Investigation:** Shaoqian Zeng, Yefang Liu.

**Methodology:** Cen Jiang, Li Wen.

**Project administration:** Li Wen, Quansheng Feng.

**Resources:** Baixue Li.

**Software:** Shaoqian Zeng, Yefang Liu, Li Wen.

**Supervision:** Baixue Li, Quansheng Feng.

**Validation:** Quansheng Feng.

**Writing – original draft:** Shaoqian Zeng.

**Writing – review & editing:** Quansheng Feng.

## Abbreviations

Abbreviations: GSG = Ganshuang granules, TCM = traditional Chinese medicine.
